# Mean Mesiodistal Width of Canine in Patients Visiting a Tertiary Care Centre: A Descriptive Cross-sectional Study

**DOI:** 10.31729/jnma.7099

**Published:** 2022-10-31

**Authors:** Radha Baral, Samarika Dahal, Sanjay Prasad Gupta

**Affiliations:** 1 Department of Oral Pathology and Forensic Dentistry, Maharajgunj Medical Campus, Maharajgunj, Kathmandu, Nepal; 2 Department of Orthodontics and Dentofacial Orthopedics, Maharajgunj Medical Campus, Maharajgunj, Kathmandu, Nepal

**Keywords:** *canine teeth*, *maxillary bone*, *sexual dimorphism*

## Abstract

**Introduction:**

The measurement of the teeth is one of the most reliable methods of identification. The teeth represent the most durable, resilient, and chemically stable part of the skeleton. The comparison of tooth dimensions is one of the tools of sex determination. Canines, in particular, are found to have the greatest degree of sexual dimorphism. The aim of the study was to determine the mean mesiodistal width of canines in patients visiting a tertiary care centre.

**Methods:**

This descriptive cross-sectional study was done among patients visiting a tertiary care centre from 25 August 2021 to 28 December 2021. The ethical approval was taken from the Institutional Review Committee [Reference number: 61 (6-11)E2078/079]. The maximum mesiodistal width of the permanent maxillary canine was measured by a digital vernier caliper. The formula given by Garn and Len was used to calculate sexual dimorphism. Convenience sampling was used. Point estimate and 95% Confidence Interval were calculated.

**Results:**

Among 104 maxillary casts studied, the mean mesiodistal width of the maxillary canine was 7.85±0.45 mm (7.76-7.93, 95% Confidence Interval). The mean mesiodistal width of the right maxillary canine in males and females were 7.90±0.48 mm and 7.83±0.45 mm respectively and that of the left maxillary canines in males and females were 7.92±0.44 mm and 7.75±0.45 mm respectively. The sexual dimorphism in the right and left maxillary canine was seen in 10 (0.96%) and 22 (2.12%) respectively.

**Conclusions:**

The mean mesiodistal width of the maxillary canine was similar to other studies done in similar settings.

## INTRODUCTION

The sex determination of the skeletal remains is a part of many medico-legal procedures. The teeth are found to be one of the accurate methods of identification. The teeth represent the most durable, resilient, and chemically stable part of the skeleton.^1,2^

One of the most researched tools for sex determination is the comparative assessment of the tooth dimensions. Canines, in particular, are witnessed to have the highest degree of sexual dimorphism.^3,4^ The proficiency of canine for sex determination is based on the influence of the Y chromosomes, which do not exhibit uniform influence on all teeth and controls the thickness of the dentin.^5,6^ Canines comparatively have less incidence of caries and periodontal diseases too.^2^ A study from Nepal have also illustrated sexual dimorphism of canine showing larger dimension in males than that in females.^7^

The objective of this study was to determine the mean mesiodistal width of canines in patients visiting a tertiary care centre.

## METHODS

A descriptive cross-sectional study was conducted in the Department of Oral Pathology and Forensic Dentistry of Tribhuwan University Teaching Hospital (TUTH) from 25 August 2021 to 28 December 2021. The ethical approval was taken from the Institutional Review Committee of the Institute of Medicine [Reference number: 61 (6-11) 078/079]. Dental casts with fully erupted morphologically well-developed permanent maxillary canines were used for the study. Dental casts with bridge or crown in canine, rotation, and overlapping, carious or restored permanent maxillary, missing, impacted canine and retained deciduous canine were excluded from the study.^7^ Convenience sampling method was used. The sample size was calculated using the following formula:


$$\begin{array}{ll} \text{n} & ={\text{Z}}^{2}\times \frac{\sigma^{2}}{{\text{e}}^{\text{2}}}\\ & =1.96^{2}\times \frac{0.72^{2}}{14^{2}}\\ & =102 \end{array}$$

Where,

n= minimum required sample size
Z= 1.96 at 95% Confidence Interval (CI)
σ = standard deviation of mean mesiodistal width of canine taken from previous study, 0.72^8^
e= margin of error, 0.14

The calculated sample size was 102. However, a total of 104 dental casts were examined.

Digital Vernier Caliper was used to measure the mesiodistal width of the permanent maxillary canine directly on the dental casts. The caliper beaks were inserted and held parallel to the long axis of the tooth. The beaks were then closed when gentle contact with a tooth was established. Garn and Lens formula was used to calculate sexual dimorphism as follows.^6^ The maximum mesiodistal width was taken into consideration and noted.

Data were entered in Microsoft Excel 2007 and analyzed using IBM SPSS Statistics 21.0. Point estimate and 95% CI were calculated.

## RESULTS

Among 104 maxillary casts studied, the mean mesiodistal width of the maxillary canine was 7.85±0.45 mm (7.76-7.93, 95% CI). The mean mesiodistal width of the right maxillary canine was 7.90±0.48 mm and 7.83±0.45 mm in males and females respectively. The mean mesiodistal width of the left maxillary canines was 7.92±0.44 mm and 7.75±0.45 mm in males and females respectively (Table 1).

**Table 1:** Mesiodistal width of the maxillary canine (n= 104).

Teeth	Sex	n (%)	Mesiodistal width Mean±SD
Right maxillary	Male	52 (50)	7.90±0.48
canine	Female	52 (50)	7.83±0.45
Left maxillary	Male	52 (50)	7.92±0.44
canine	Female	52 (50)	7.75±0.45

The mean age of the patients was 20.78±6.021 years. The mean age of males and females were 20.90±5.417 and 21.20±5.911 years respectively. The sexual dimorphism in the right maxillary canine was 10 (0.96%) while in the left maxillary canine was 22 (2.12%).

## DISCUSSION

The mean mesiodistal width of the permanent maxillary canine in the present study was 7.85 mm which was similar to other studies.^9,10^ The mean mesiodistal width of the permanent maxillary canine in the present study was similar on both sides of the jaw in agreement with other studies.^11,12^ However, few studies have reported a significant difference between mesiodistal width of canines in both sexes.^4,13-16^

The present study revealed sexual dimorphism in the right and left maxillary canines to be less than in other studies. In the Nigerian population, the right maxillary canine showed 4.58% of sexual dimorphism while the left-sided canine revealed 3.85%.^2^ Nepalese student-based studies have reported sexual dimorphism of 3.2% and 4.4% on the right and left sides respectively.^7^ Similarly another Indian population-based study has reported sexual dimorphism in canines on the right and the left side to be 3.31% and 3.29% respectively.^17^ Similarly, Gujrati population-based study in India has reported sexual dimorphism percentage on the right side to be 8.87% and left side to be 7.26%.^1^ However, another Indian population-based study found 5.5% of sexual dimorphism in mesiodistal width of maxillary canine.^14^ A study done on the Malaysian population revealed 8.5% and 6.5% dimorphism on the right and left sides respectively.^3^ Multivariate discriminant analysis in the Saudi population demonstrated 65.5% correct discrimination of sexes when the values of right and left maxillary canine were combined.^18^

These variations in different studies might be due to the diversity in the odontometric features among individuals from different populations. Also, the influence of environmental, cultural, racial and biological factors may contribute to dissimilarity.^3,19^ The complex interaction of the assorted genetic and environmental factors leads to inequality in the extent of dimorphism.^19^

This study was conducted in a single centre among small sample size so the study might not be generalizable to a larger population. The influence of different factors was not evaluated as this was a cross-sectional study.

## CONCLUSIONS

The mean mesiodistal width of the maxillary canine was similar compared to other studies done in similar settings. These findings can be important for sex determination based on canines.
